# Synergistic effects and mechanisms of basalt fibers and polycarboxylate superplasticizer on cement–fly ash stabilized aeolian sand and crushed stones

**DOI:** 10.1371/journal.pone.0327351

**Published:** 2025-07-15

**Authors:** Jie Liu, Qinli Liu, Chao Pu, Chunsheng Zhu, Yanhong Li, Junjie Zhou, Yan Xu

**Affiliations:** 1 Xinjiang Transportation Planning, Survey and Design Institute Co., Ltd, Urumqi, Xinjiang, China; 2 Xinjiang Key Laboratory for Safety and Health of Transportation Infrastructure in Alpine and High-altitude Mountainous Areas, Urumqi, Xinjiang, China; 3 College of Civil Engineering and Architecture Xinjiang University, Urumqi, Xinjiang, China; 4 Xinjiang Communications Investment Construction Management Co., Ltd, Urumqi, Xinjiang, China; 5 Xinjiang Kusha Highway Development Co., Ltd, Aksu, Xinjiang, China; 6 Yili Communications Investment Group Co., Ltd, Yining, Xinjiang, China; Shandong University of Technology, CHINA

## Abstract

To enhance the mechanical and durability properties of cement-fly ash stabilized aeolian sand and crushed stones, the synergistic optimization effects of basalt fibers and polycarboxylate superplasticizer were investigated. First, two full factorial experiments were conducted to evaluate the individual and combined effects of basalt fiber volume content and polycarboxylate superplasticizer mass content. Then, four mix proportions were selected to verify the durability optimization. Finally, SEM, EDS, and XRD were used to elucidate the underlying micro-mechanisms. The results indicate that the optimal combination was 0.1% volume content of 12-mm-long basalt fibers and 1.0% mass content of polycarboxylate superplasticizer, which yielded a compressive strength of 13.3 MPa and a splitting tensile strength of 1.14 MPa at 28 days. Compared to the control group and individual addition of basalt fibers or polycarboxylate superplasticizer, the group with both basalt fibers and polycarboxylate superplasticizer had 33.00%, 16.67%, and 14.66% higher compressive strength and 52.00%, 31.03%, and 28.09% higher splitting tensile strength, respectively. Furthermore, the combined optimization improved the durability, decreased the thermal shrinkage by 49.85%, 32.35%, and 28.84%, and decreased the drying shrinkage by 68.95%, 33.15%, and 47.58%. The micro-experiments demonstrate that the bridging effect of basalt fibers during micro-crack formation and the synergistic action of polycarboxylate superplasticizer enhanced the uniformity and density of the mixture and that they are the primary factors that contribute to the strength development. Therefore, cement-fly ash stabilized aeolian sand and crushed stones can be optimized by using basalt fibers and polycarboxylate superplasticizer.

## 1. Introduction

The unique geographical environment of Xinjiang, China, presents significant challenges to the construction of desert highways due to the scarcity of high-quality road construction materials [1,2]. Xinjiang boasts expansive deserts, including the Taklamakan and Gurbantunggut, with abundant reserves of wind-blown sand. So far, the region’s use of this resource has been limited to small-scale engineering material substitutions, but its vast potential for wider applications remains largely unexplored. Hence, there is an urgent need for practitioners to adopt innovative methods to utilize local aeolian sand and enhance its applicability and durability for highway pavements in Xinjiang [3,4]. Both domestic and international scholars have conducted preliminary research on aeolian sand. In China, aeolian sand is classified as A-3 according to the soil classification system of the AASHTO (American Association of State Highway and Transportation Officials), which indicates its potential as an excellent road construction material. Nzuki et al. [5] analyzed the major elements and mineral characteristics of aeolian sand samples from the Tengger Desert using X-ray fluorescence, electron probe micro-analysis, and X-ray diffraction techniques and demonstrated the potential of aeolian sand as an industrial engineering material. Countries in Europe, America, and the Middle East have successfully applied aeolian sand in highway construction. Elipe and Lopez-Querol [6] found that aeolian sand exhibited characteristics such as non-plasticity and uniform particle size, which make it highly suitable for infrastructure development. Their research also explored various methods to enhance aeolian sand and addressed its limitations to establish a solid foundation for its widespread application. Additionally, Lopez-Querol et al. [7] effectively improved the engineering properties of aeolian sand in Jeddah, Saudi Arabia, through cement stabilization, which makes it more suitable for practical engineering applications. Netterberg and Elsmere [8] conducted a 50-year field observation of the aeolian sand base layer of a main road in South Africa and provided valuable data that could inform the use of aeolian sand materials in China.

The fine particles and smooth, rounded shape of aeolian sand result in weak adhesion with other materials, which adversely affects the strength of the material. The poor gradation of aeolian sand contributes to its insufficient load-bearing capacity after compaction. The high salt content in aeolian sand may also cause chemical reactions with water, which impacts its durability. Consequently, aeolian sand can be used as an engineering material in specific applications, but it requires appropriate treatment and enhancement prior to use [9]. The stabilization of aeolian sand typically involves both chemical and physical reinforcement methods [10].

Common methods for chemical stabilization involve using inorganic binders (such as cement-based stabilizers, geopolymer stabilizers, and lime-fly ash stabilizers) or curing agents to reinforce aeolian sand. Sheng and Li et al. [11,12] used cement to solidify aeolian sand and showed that incorporating 6% cement in the sand with 28 days of thermal shrinkage resulted in an average compressive strength of 0.156 MPa. Aeolian sand concrete with a cement: crushed stone: aeolian sand mix ratio of 11.47%: 27.98%: 60.55% achieved a compressive strength of 2.32 MPa. Further research [13] indicates that when the cement content was 11%, the 7-day unconfined compressive strength of the cement-stabilized crushed stone and aeolian sand base layer satisfied high-grade highway construction standards. Bai et al. [14] studied fly ash and blast furnace slag-based geopolymer-stabilized aeolian sand and achieved strengths of 6 MPa and 9.5 MPa at 7 days and 28 days, respectively. Jales Silva et al. [15] investigated the performance of ceramic polishing waste and lime-stabilized aeolian sand at optimal mix ratios and obtained 28-day compressive and splitting strengths of 1.56 MPa and 0.16 MPa, respectively. Kamon et al. [16] and Toraldo et al. [17] concluded that cement-fly ash mixtures were suitable for highway subgrade applications. Liu et al. [18] demonstrated that with 66% aeolian sand content, the 7-day unconfined compressive and splitting strengths were 2.8 MPa and 0.61 MPa, respectively. Thus, 100% substitution of fine aggregate in cement-fly ash stabilized crushed stone with aeolian sand can satisfy highway subgrade design requirements. Sun et al. [19] found that using 9% DHT soil curing agent to solidify aeolian sand resulted in 7-day and 28-day compressive strengths of 3.3 MPa and 6.4 MPa, respectively.

Although cement is cost-effective, using high amounts of cement for reinforcement can lead to cracks due to drying shrinkage and thermal shrinkage, which would compromise the overall stability and durability. Additionally, a high cement content corresponds to significant amounts of CO_2_ during application and contributes to the greenhouse effect. Although geopolymer and lime-fly ash composites can use recycled waste materials, they often fail to satisfy the high load-bearing requirements of specific projects. Commercially available chemical curing agents can provide excellent stabilization and durability, but their high costs are a significant drawback. Consequently, many researchers have explored physical reinforcement methods.

Physical reinforcement typically involves the use of various fibers such as polypropylene, glass, and basalt fibers to enhance the strength of aeolian sand. Belferrag et al. [20] demonstrated that the physical properties, orientation distribution, content, and bonding strength between the fibers and the matrix significantly influenced the compressive strength of fiber-reinforced aeolian sand concrete. Boulekbache et al. [21] corroborated that the fiber orientation had critical impact on the concrete compressive strength. Jiang et al. [22] indicated that the improvement of fiber dispersion, refinement of the pore structure, and enhanced performance of ultra-high-performance concrete made from aeolian sand are closely interconnected. Zhang et al. [23] and Hu et al. [24] revealed that the incorporation of polypropylene fibers increased the tensile strength and crack resistance of cement-stabilized and lime-fly ash-stabilized aeolian sand. Zhang et al. [25,26] found that glass fibers improved the interface connection between cement-stabilized aeolian sand mixtures and fibers, which formed an internal three-dimensional network structure with enhanced resistance to damage and deformation. Ruan [27–30] discovered that with a basalt fiber content of 0.8%, both unconfined compressive strength and freeze-thaw resistance maximized. Dong et al. [31,32] reported that at a basalt fiber content of 1.5 kg/m³, the strength enhancement ratios of basalt fiber cement-modified aeolian sand samples were 1.14–1.54, and the ductility enhancement ratio was 1.43–2.67. To optimize the composite performance between basalt fibers and inorganic binders, the surface of basalt fibers can be modified using silane coupling agents. These agents increase the surface roughness of the fibers, thereby enhancing the interfacial friction between the fibers and the cementitious matrix, which in turn improves the bonding performance of the composite material [33]. España et al. [34] used silane coupling agents to modify the surface of basalt fibers and reported significant improvements in the mechanical properties of the resulting composites. Compared to untreated basalt fibers, the modified fibers exhibited a maximum tensile modulus of up to 18.6 GPa, representing a 250% increase. In addition, Li et al. [35] treated basalt fibers with 8% KH-550 silane coupling agent, resulting in a fiber dispersion rate of 81.625% in the cement matrix, indicating a favorable modification effect.

In conclusion, the use of cement-fly ash-stabilized aeolian sand is a viable method to reduce CO_2_ emissions and recycle fly ash. However, its strength may not meet all engineering requirements. However, the arid and highly variable climate in Xinjiang directly results in a decrease in the crack resistance of the material. Therefore, researchers often use water-reducing agents to reduce moisture evaporation by lowering capillary pressure, improving the uniform distribution of water, and forming a dense internal structure, thereby enhancing the material’s crack resistance.

Miao et al. [36] demonstrated that polycarboxylate superplasticizers effectively enhanced the pore structure of cement-based materials through their molecular structure, grafting groups, and grafting mass. Li et al. [37] found that these superplasticizers provided high dispersibility and retention, shortened the initial induction period, and prolonged the induction phase of cement hydration, i.e., they exhibited a retardation effect. Chen et al. [38] showed that polycarboxylate superplasticizers could reduce the peak heat of hydration, inhibit the crystallization of Ca(OH)2 and AFt, increase the slurry density, and decrease the content of chemically bound water. Li et al. [39] used a self-synthesized early-strength polycarboxylate superplasticizer to increase the 7-day strength by 8 MPa, mitigate the risk of chloride ion corrosion and likelihood of alkali-silica reactions, and consequently improve the concrete durability. Zhang et al. [40] found that the addition of polycarboxylate superplasticizers to a ternary composite system of slag-desulfurized gypsum-sulphoaluminate cement resulted in a maximal 28-day compressive strength of 75.09 MPa. It can be observed that, these superplasticizers can delay the overall hydration process of cementitious materials, reduce the rates of thermal and drying shrinkage, and enhance the crack resistance of concrete. However, while water-reducing agents slow down the initial hydration rate, they also prolong the hydration process, leading to extended setting times. This effect is particularly pronounced in low-temperature environments, where the delay in setting time is amplified, further inhibiting the early strength development of cement-based materials.

The chemical stabilization of aeolian sand results in high strength but often incurs durability issues, whereas physical stabilization provides excellent durability at the expense of strength. Meanwhile, the use of water-reducing agents alone may inhibit the early mechanical strength of cement-based materials, especially in low-temperature environments. However, ensuring the crack resistance and mechanical performance of aeolian sand subgrade under extreme conditions is crucial for cost reduction and efficiency in desert highway construction. Therefore, it is necessary to conduct more in-depth research on aeolian sand-stabilized subgrades that combine excellent mechanical and crack resistance properties.

Thus, the objective of this study is to develop an aeolian sand base that combines excellent mechanical and crack-resistant properties using a combination of physical and chemical modifications. Firstly, the orthogonal test was conducted to study the effects of different basalt fiber contents and polycarboxylate superplasticizer dosages on the mechanical properties of cement-fly ash stabilized aeolian sand-gravel base. Secondly, the durability of the cement-fly ash stabilized aeolian sand-gravel base with combined incorporation of basalt fibers and polycarboxylate superplasticizers was verified. Next, XRD (X-ray diffraction), SEM (scanning electron microscopy), and EDS (energy dispersive spectroscopy) were used to analyze the mechanisms of strength development at the micro level for the combined incorporation of basalt fibers and polycarboxylate superplasticizers. Finally, the optimal mix ratios and dosages for single incorporation of basalt fibers, single incorporation of polycarboxylate superplasticizers, and combined incorporation of basalt fibers and polycarboxylate superplasticizers were identified.

The findings of this research aim to facilitate the high-quality, extensive, and multi-level application of aeolian sand in road engineering.

## 2. Materials and methods

### 2.1. Cement

The cement was PO42.5 ordinary Portland cement, and all performance indicators satisfy the standard requirements of GB 175–2023. Table 1 shows the technical parameters, including fineness, water requirement for standard consistency, coagulation time, flexural strength, compressive strength, and soundness.

**Table 1:** Portland cement technical parameters.

Fineness (80 μm square-hole sieve)/%	Water required for standard consistency/%	Setting time/min		Flexural strength/MPa		Compressive strength/MPa		Soundness
		Initial setting	Final setting	3 d	28 d	3 d	28 d	
7.2	28.6	181	214	5.3	7.6	29.5	49.4	Qualified

### 2.2. Fly ash

The fly ash was Class-F, Grade-II fly ash, and all performance indicators comply with the standard requirements of GB/T 1596–2017. Table 2 shows the technical parameters, including fineness, water demand ratio, loss on ignition, water content, free calcium oxide mass fraction, and total mass fraction of SiO_2_, Al_2_O_3_, and Fe_2_O_3_.

**Table 2:** Technical parameters of Class-F Grade-II fly ash.

Fineness (40 μm square-hole sieve)/%	Water demand ratio/%	Loss on ignition/%	Water content/%	Free calcium oxide mass fraction/%	Total mass fraction of SiO_2_, Al_2_O_3_, and Fe_2_O_3_/%
19.6	90	1.8	0.04	0.5	78.6

**Fig 1:**
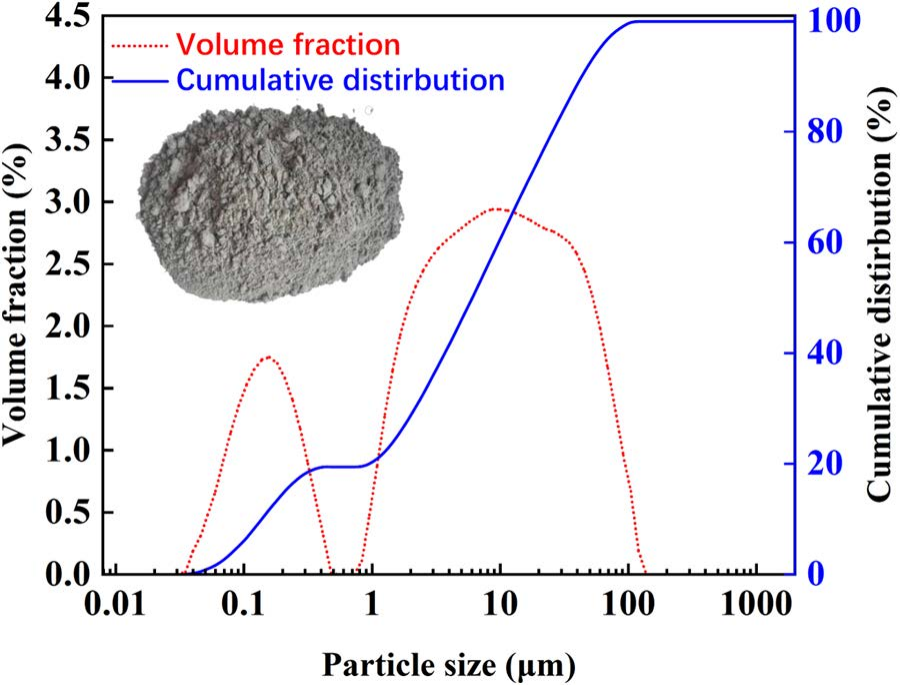
Fly ash appearance, particle size distribution.

**Fig 2:**
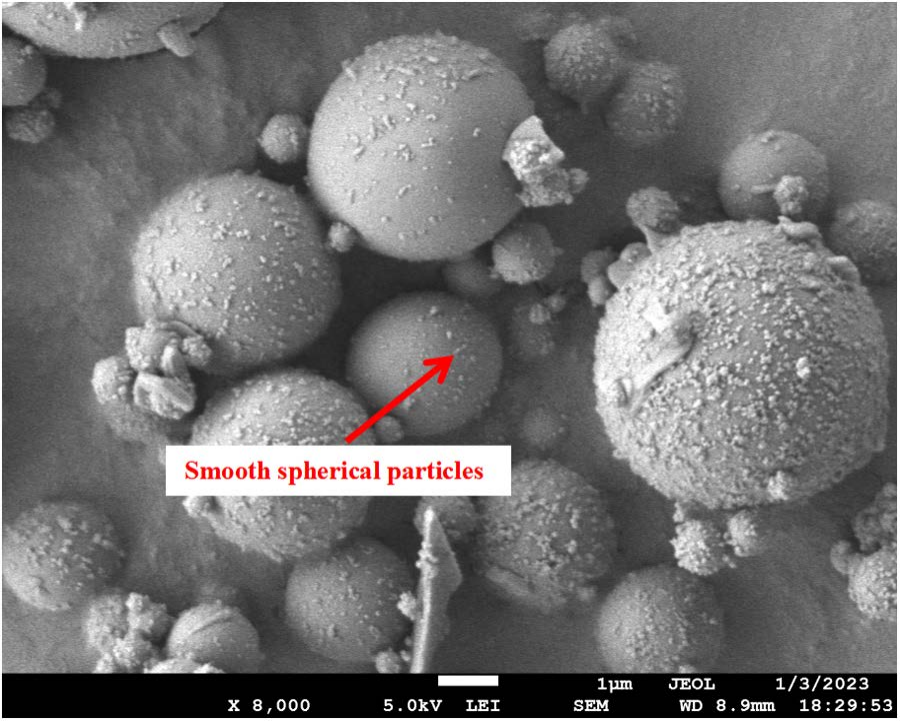
Fly ash SEM.

Fig 1 shows that the fly ash appeared as a light gray powder. According to the results from the laser diffraction particle size analyzer, the particle size range of the fly ash was approximately 0.035–140 μm, and the volume average particle size was 16.888 μm. The particle size distribution curve shows peaks at 0.035–0.55 μm and 0.8–140 μm, which indicates a higher quantity of fly ash particles in these size ranges. SEM testing was conducted on the fly ash in the experiment. Fig 2 shows that the fly ash particles were closely packed and mostly smooth-surfaced spherical particles. Larger fly ash particles had rough surfaces, and smaller particles adhered to the surfaces of larger particles.

### 2.3. Limestone crushed stone

The selected crushed stone was limestone, and all performance indicators satisfied the requirements of the JTG 3432−2024 specification. Table 3 shows the specific technical parameters, including particle size, apparent density, crushing value, mud content, water absorption, and needle-like content.

**Table 3:** Technical parameters of limestone crushed stone.

Particle size/mm	Apparent density/(kg/cm^3^)	Crushing value/%	Mud content/%	Water absorption/%	Needle-like content/%
9.5–19	2688	10.4	0.55	0.68	3.3
19–31.5	2673	10.9	0.28	0.53	2.8

### 2.4. Aeolian sand

Aeolian sand was collected from the moving sand dunes at the northern edge of the Gurbantünggüt Desert, where sand particles comprised 96.03% of the total weight. The sand had Cu (coefficient of uniformity) =2.1 and Cc (coefficient of curvature) =0.9, which indicates a relatively poor gradation and finer particles. Table 4 lists the specific technical parameters, including apparent density, moisture content, mud content, sulfate content, sulfate content, Cu, and Cc.

**Table 4:** Technical parameters of aeolian sand.

Apparent density/kg/cm^3^	Moisture content/%	Mud content/%	Sulfate content/%	Cu	Cc
2.652	1.75	1.4	0.13	2.1	0.9

**Fig 3:**
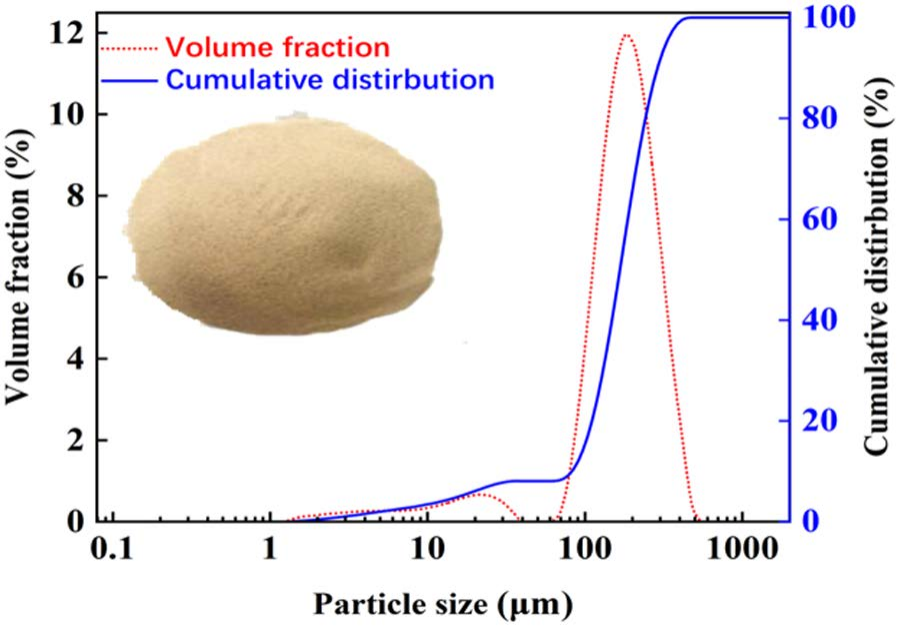
Appearance, particle size distribution.

Fig 3 shows that the sand particles were light yellow in color. Data from the laser diffraction particle size analyzer indicate that the particle size of the sand was approximately 1.25–550 μm, with a volume average particle size of 200.43 μm. The particle size distribution curve peaked at 1.25–40 μm and 60–550 μm, which indicates a higher quantity of sand particles in these size ranges.

**Fig 4:**
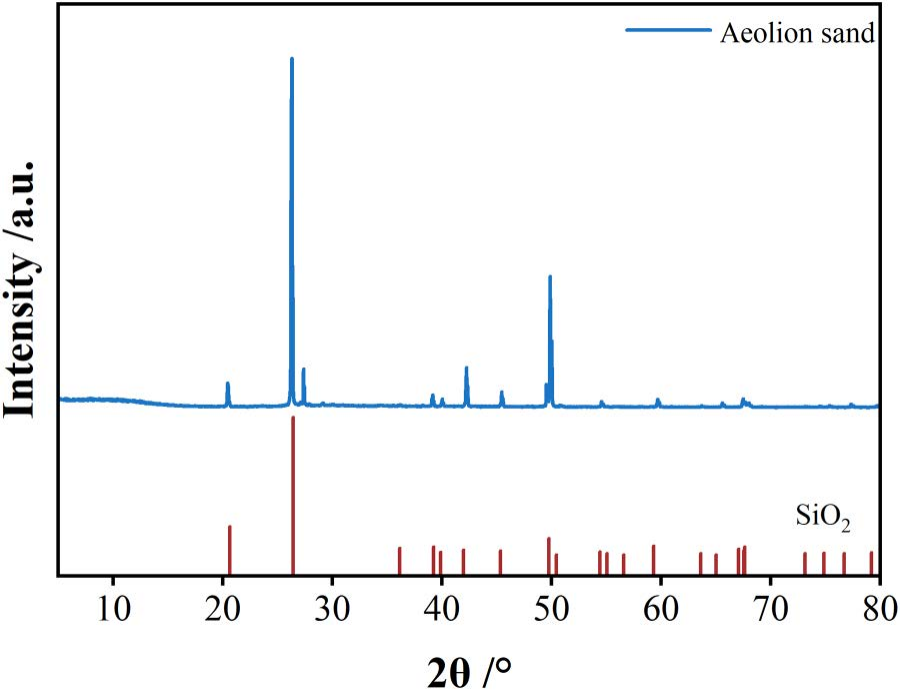
Aeolian sand SEM.

**Fig 5:**
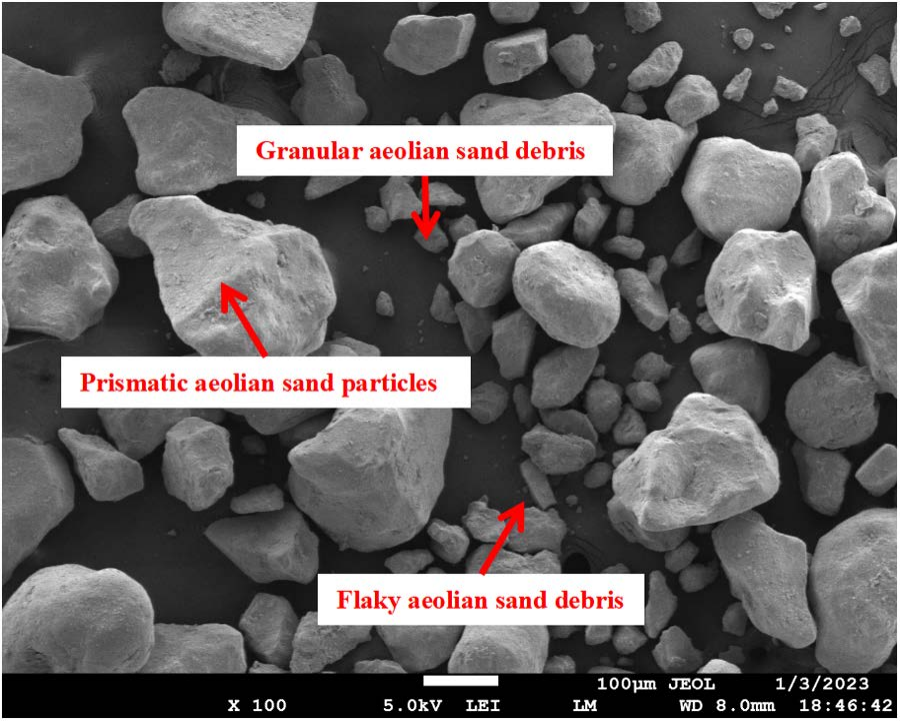
XRD result of aeolian sand.

A comparison of the aeolian sand XRD (Fig 4) with standard PDF cards determined that the main components of the aeolian sand were SiO_2_. SEM results (Fig 5) show that the sand particles were mainly columnar, granular, and flaky in shape with obvious pores between particles and a loose structure. Larger particles exhibited point-to-point contact and interlocking characteristics, and smaller particles acted as fillers for the gaps among the larger particles.

### 2.5. Basalt fiber

**Fig 6:**
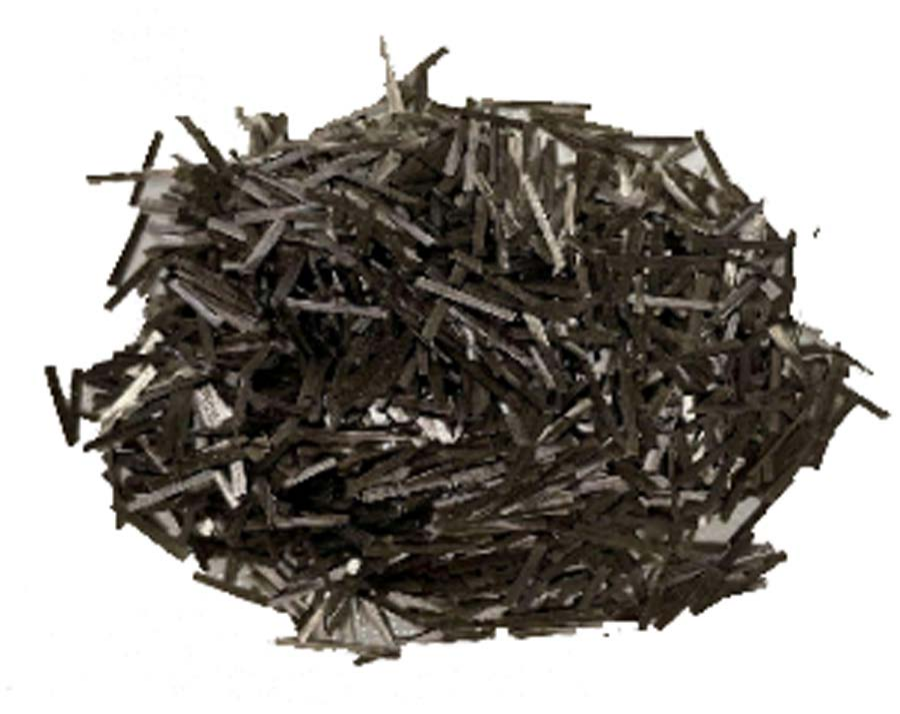
Sample image of the 12 mm basalt fiber.

The test used basalt fibers with short cut lengths of 6, 9, 12, 15, and 18 mm and aspect ratios of 400, 600, 800, 1000, and 1200. Fig 6 shows the dimensional morphology of the 12 mm basalt fiber. Table 5 shows the specific technical parameters, including length, length-to-diameter ratio (L/D), density, elastic modulus, tensile strength, and elongation at break.

**Table 5:** Technical parameters of the basalt fibers.

Length/mm	L/D ratio	Density/(kg/m^3^)	Elastic modulus/GPa	Tensile strength/MPa	Elongation at break/%
6	400	2650	91–10	3000–4800	3.1
9	600	2650	91–110	3000–4800	3.1
12	800	2650	91–110	3000–4800	3.1
15	1000	2650	91–110	3000–4800	3.1
18	1200	2650	91–110	3000–4800	3.1

### 2.6. Polycarboxylate superplasticizer

**Fig 7:**
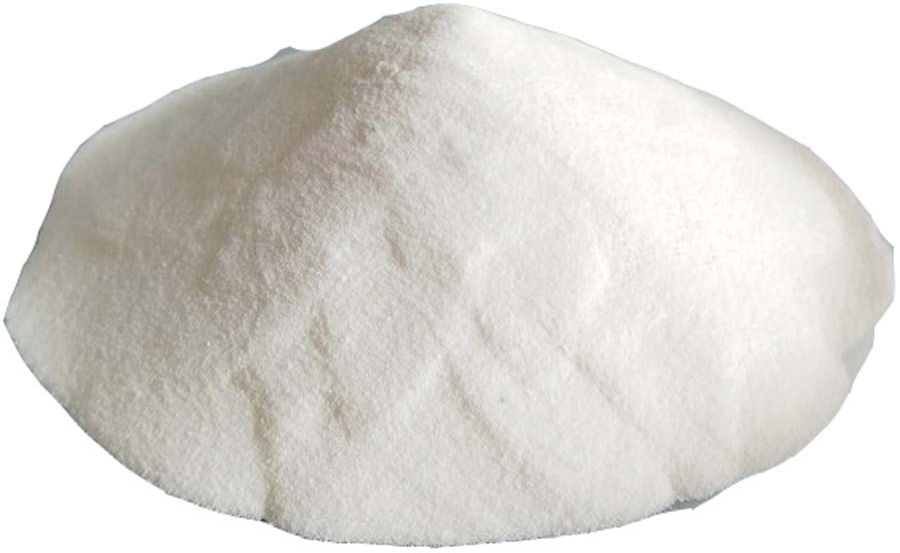
Polycarboxylate superplasticizer.

The water-reducing agent was a polycarboxylate powder with a water reduction rate of 27%. Fig 7 shows its morphology.

### 2.7. Overall procedure

**Fig 8:**
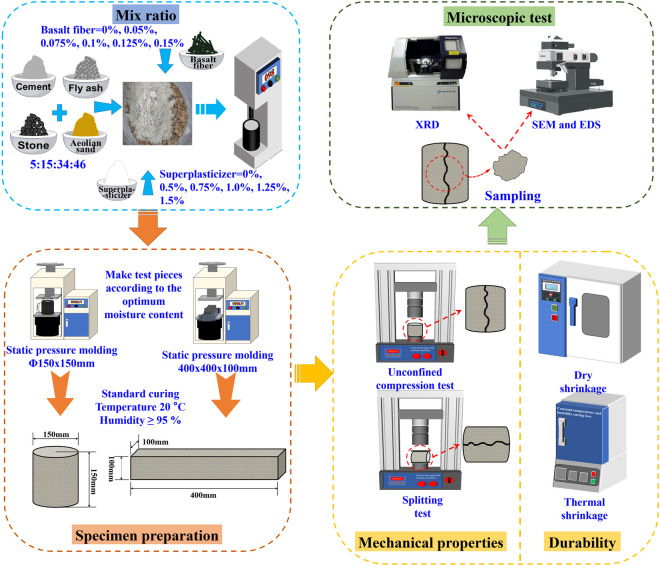
Overall process of test.

Based on a fixed cement: fly ash: crushed stone: aeolian sand mix ratio of 5:15:34:46, different amounts of basalt fibers and polycarboxylate superplasticizers were added. Macro and micro experiments were conducted to systematically study the performance of the fiber-superplasticizer cement-fly ash stabilized aeolian sand and crushed stone base layer. On the macro scale, the mechanical properties and durability of the aeolian sand were verified. On the micro scale, XRD, SEM, and EDS were used to elucidate the strength development mechanisms. Ultimately, the optimal mix ratios and dosages for single incorporation of basalt fibers, single incorporation of polycarboxylate superplasticizers, and combined incorporation of basalt fibers and polycarboxylate superplasticizers were identified. Fig 8 illustrates the technical route and main experimental process of this study. All the aforementioned tests were carried out at Xinjiang Jiaokan Zhiyuan Engineering Technology Co., Ltd.

### 2.8. Test methods

#### 2.8.1. Modified proctor compaction test.

The modified compaction proctor test was conducted according to JTG 3441−2024 (T0804-1994) method. Initially, five different and consecutive moisture contents were preset. Then, based on the actual dry density and actual moisture content obtained from the compaction test at each preset moisture level for each mixture ratio, a moisture content versus dry density curve was plotted. Finally, a quadratic curve fitting was applied to determine the optimum moisture content (OMC) and maximum dry density (MDD) for each group of mixture ratios, as shown in the Table 6.

**Table 6:** OMC and MDD for different mixture ratios.

Mixture ratios	OMC	MDD	Mixture ratios	OMC	MDD
B0P0	2.175	5.9	B0P1	2.166	5.4
B1P0	2.169	5.9	B1P1	2.170	5.6
B2P0	2.169	5.8	B2P1	2.167	5.7
B3P0	2.165	6.0	B3P1	2.177	5.5
B4P0	2.173	6.0	B4P1	2.173	5.6
B5P0	2.171	5.9	B5P1	2.173	5.5
B0P2	2.197	5.2	B0P3	2.198	5.2
B1P2	2.201	5.2	B1P3	2.228	5.2
B2P2	2.203	5.2	B2P3	2.203	5.3
B3P2	2.185	5.5	B3P3	2.184	5.4
B4P2	2.178	5.7	B4P3	2.169	5.4
B5P2	2.183	5.5	B5P3	2.199	5.2
B0P4	2.193	5.1	B0P5	2.205	5.1
B1P4	2.194	5.1	B1P5	2.207	5.1
B2P4	2.187	5.2	B2P5	2.217	5.1
B3P4	2.192	5.1	B3P5	2.193	5.0
B4P4	2.192	5.1	B4P5	2.189	4.9
B5P4	2.184	5.0	B5P5	2.195	4.9

#### 2.8.2. Mechanical properties.

The mechanical performance tests were conducted according to the JTG 3441−2024 standard using methods T0805-1994 and T0806-1994. Cylindrical specimens with dimensions of φ150 mm × 150 mm were prepared using the static compaction method. According to the specification, 13 parallel specimens are typically required for each test group. However, due to the large number of test combinations in this study, 6 parallel specimens were prepared for each group. All test results met the requirement of a coefficient of variation (CV) less than 20%, ensuring data reliability. Based on preliminary experimental studies, a full factorial design was used with basalt fibers of 12-mm length at varying volume contents (B0 = 0%, B1 = 0.05%, B2 = 0.075%, B3 = 0.1%, B4 = 0.125%, B5 = 0.15%) and polycarboxylate superplasticizers at different mass contents (P0 = 0%, P1 = 0.5%, P2 = 0.75%, P3 = 1.0%, P4 = 1.25%, P5 = 1.5%). These dosage intervals were selected based on their favorable mechanical performance observed in the preliminary tests. The factorial design is summarized in Table 7. Then, additional full factorial design was conducted with basalt fibers of varying lengths (6, 9, 12, 15, and 18 mm) at a volume content of B3 = 0.1% and polycarboxylate superplasticizers at mass contents of P0 = 0% and P3 = 1.0%. Table 8 shows the full factorial design table for this experiment. The curing periods for the specimens were set at 7, 14, and 28 days.

**Table 7:** Orthogonal design 1.

	B0(0%)	B1(0.05%)	B2(0.075%)	B3(0.1%)	B4(0.125%)	B5(0.15%)
P0 (0%)	B0P0	B1P0	B2P0	B3P0	B4P0	B5P0
P1 (0.5%)	B0P1	B1P1	B2P1	B3P1	B4P1	B5P1
P2 (0.75%)	B0P2	B1P2	B2P2	B3P2	B4P2	B5P2
P3 (1.0%)	B0P3	B1P3	B2P3	B3P3	B4P3	B5P3
P4 (1.25%)	B0P4	B1P4	B2P4	B3P4	B4P4	B5P4
P5 (1.5%)	B0P5	B1P5	B2P5	B3P5	B4P5	B5P5

**Table 8:** Orthogonal design 2.

	6mmB3 (0.1%)	9mmB3 (0.1%)	12mmB3 (0.1%)	15mmB3 (0.1%)	18mmB3 (0.1%)
P0 (0%)	6mmB3P0	9mmB3P0	12mmB3P0	15mmB3P0	18mmB3P0
P3 (1.0%)	6mmB3P3	9mmB3P3	12mmB3P3	15mmB3P3	18mmB3P3

#### 2.8.3. Crack resistance.

According to test methods T0855-2009 and T0854-2009 in specification JTG 3441−2024, 100 mm × 100 mm × 400 mm specimens were prepared to evaluate the crack resistance of four mixtures: B0P0, B0P3, B3P0, and B3P3. For the thermal shrinkage test, the samples were placed in a climate chamber and cycled through six temperature levels from 40°C to −20°C at a rate of 0.5°C/min for 3 h. The dial gauge reading was read 5 min before the end of each cycle to calculate the thermal shrinkage strain and coefficient. Three parallel specimens were tested for each group, and the measurement errors were within the acceptable limits specified in the standard. For the dry shrinkage test, the samples were placed in a dry shrinkage box, and the dial gauge readings and specimen masses were recorded at specified intervals over 28 days to determine the water loss rate, dry shrinkage, strain, and shrinkage coefficient. Six parallel specimens were used for each group, and the results also met the error tolerance requirements of the specification.

#### 2.8.4. Microscopic experiments.

Using XRD, SEM, and EDS, scans were performed on 7- and 28-day-cured samples of B0P0, B0P3, B3P0, and B3P3. Phase analysis was obtained from these scans. The microstructure, structure of hydration reaction compounds, and elements in the regions of the microstructure were observed and analyzed.

## 3. Results and discussion

### 3.1. Mechanical properties test

#### 3.1.1. Unconfined compressive strength test.

**Fig 9:**
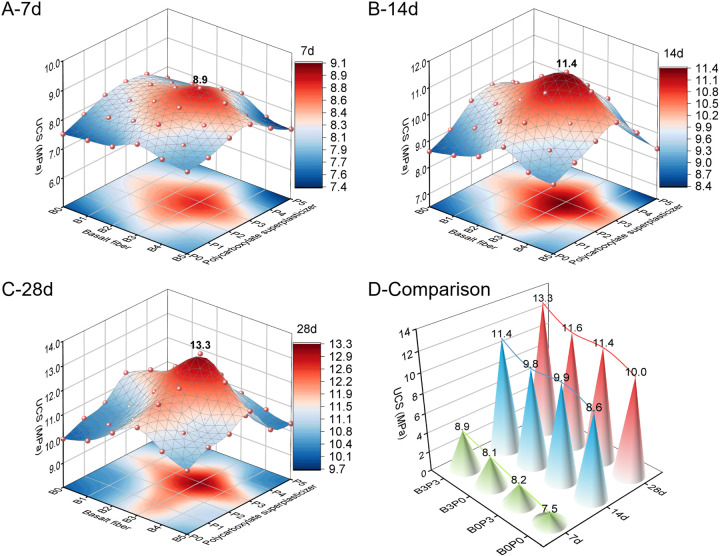
Unconfined compressive strength.

Fig 9 shows that with a constant dosage of polycarboxylate superplasticizer or basalt fiber content, the UCS (unconfined compressive strength) of the mixtures at each curing age initially increased and subsequently decreased when the content of the single additive increased. The highest strength for all mixing ratio groups was achieved at levels B3 and P3. When the curing time increased, the UCS for each mix ratio gradually increased. The UCS values for B3P3 at 7, 14, and 28 days were 8.9, 11.4, and 13.3 MPa, respectively. This trend can be attributed to the optimal amount of basalt fibers being uniformly dispersed in the mixture, which enhanced the bonding between fibers and cementitious materials and improved the UCS. However, excessive fiber content may lead to agglomeration, create weak points in the internal structure, and reduce the overall strength [41]. Additionally, an appropriate quantity of polycarboxylate superplasticizer facilitates the dispersion of cementitious particles and accelerates the hydration reaction, which enhances the strength of the mixture. Conversely, an excess of superplasticizer can result in segregation and bleeding, which increases the porosity of the mixture and consequently diminishes its strength.

During the 7-, 14-, and 28-day curing periods, the combined addition of basalt fiber and polycarboxylate superplasticizer (B3P3) resulted in the highest increases in compressive strength of 33%, 16.67%, and 16.33% compared to the baseline group (B0P0), single addition of superplasticizer (B0P3), and single addition of basalt fiber (B3P0), respectively. The physical reinforcement provided by basalt fibers and enhanced hydration efficiency from the polycarboxylate superplasticizer complemented each other and promoted a denser and more uniform internal structure of the mixture. Notably, the improvement rate in UCS for the combined addition group increased from 18.67% (7 days) to 33% (28 days) relative to the control group. The single-addition mixtures (B0P3, B3P0) also improved in performance but to a lesser extent than the combined group (B3P3). For example, during the 28-day curing period, the UCS values for B0P3 and B3P0 increased by 14% and 16%, respectively, compared to B0P0, both of which were lower than the improvement of B3P3. These data clearly indicate that although the single addition of basalt fiber or polycarboxylate superplasticizer can enhance the performance of the mixture, the combined incorporation of both additives yields superior strength increase. This finding confirms the effectiveness of the combined strategy in enhancing the performance of the mixture.

#### 3.1.2. Splitting tensile strength test.

**Fig 10:**
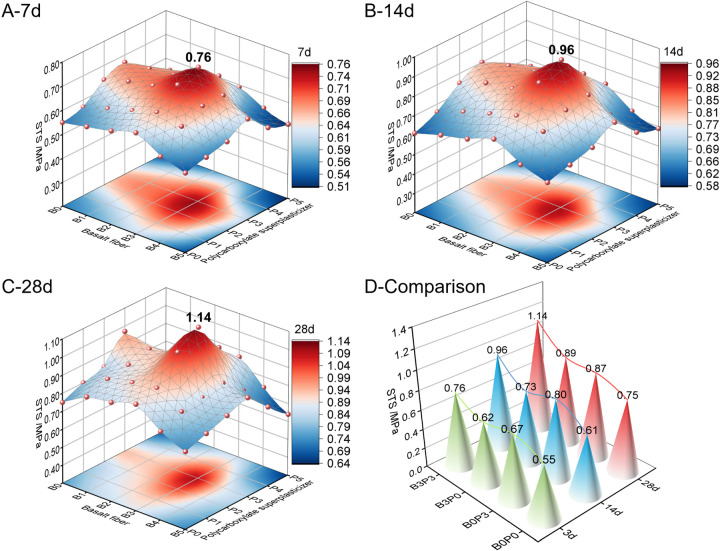
Splitting tensile strength.

Fig 10 illustrates that with a constant dosage of polycarboxylate superplasticizer or basalt fiber, the STS (splitting tensile strength) of the mixtures at each curing age initially increases and subsequently decreases when the content of the single additive increases. The highest strength across all mixing ratio groups was observed at levels B3 and P3. When the curing time increased, the STS of each mix ratio gradually improved. Specifically, the recorded STS for B3P3 at 7, 14, and 28 days was 0.76, 0.96, and 1.14 MPa, respectively.

During the 7-, 14-, and 28-day curing periods, the STS of the baseline combination (B0P0) increased from 0.55 MPa at 7 days to 0.75 MPa at 28 days, i.e., an increase rate of 36.36%. The STS of the combination that contained only basalt fibers (B3P0) increased from 0.62 MPa to 0.89 MPa over the same duration, i.e., an increase rate of 43.55%. In contrast, the combination with only polycarboxylate superplasticizer (B0P3) increased from 0.67 MPa to 0.87 MPa with an increase rate of 29.85%. Although B0P3 had a lower increase rate than B3P0, the result demonstrates the positive impact of the superplasticizer on the concrete performance. Notably, the combined addition (B3P3) demonstrated the most significant improvement in STS across all curing periods from 0.76 MPa at 7 days to 1.14 MPa at 28 days, i.e., a remarkable increase rate of 50.00%. This significant rate clearly exceeds the effects of adding only basalt fibers or polycarboxylate superplasticizer. During the 28-day curing period, the STS of B3P3 was approximately 52.00%, 31.03%, and 28.09% higher than that of B0P0, B0P3, and B3P0, respectively. These comparative results emphasize the substantial improvement in STS due to the combined addition of basalt fibers and polycarboxylate superplasticizer, which likely occurred because basalt fibers had a bridging effect during the micro-crack formation and the superplasticizer improved the uniformity and density of the concrete. These findings confirm the effectiveness of the combined addition strategy in enhancing the performance of the mixture.

#### 3.1.3. Determination of basalt fiber length.

**Fig 11:**
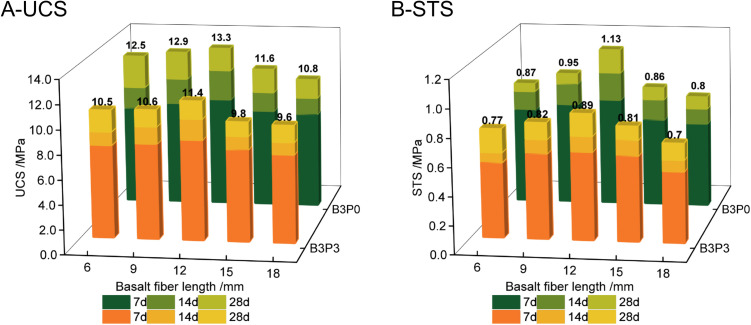
Relationship between basalt fiber length and strength.

As shown in Fig 11, for the optimal basalt fiber content (B3), both UCS and STS of the mixture first increased and subsequently decreased with increasing fiber length. The strengths maximized at a fiber length of 12 mm. This trend suggests that at the optimal length of 12 mm, the fibers can establish the most effective network structure in the matrix. This optimal network enhances the bonding strength of the mixture, its overall strength, its resistance to cracking, and its ability to distribute loads. However, when the fiber length exceeds this optimal range, issues such as entanglement and poor dispersion can arise. These complications may create weak points in the mixture and disrupt the bond between aggregates and cement paste, which ultimately reduces the UCS and STS. These findings are consistent with the results reported by Zhang [41] and confirm that a fiber length of 12 mm is optimal for basalt fibers.

### 3.2. Durability test

To study the durability performance of the mixture specimens with mix ratios B0P0, B0P3, B3P0, and B3P3, the average thermal shrinkage coefficient and the average drying shrinkage coefficient were calculated based on thermal shrinkage and drying shrinkage tests.

#### 3.2.1. Thermal shrinkage.

**Fig 12:**
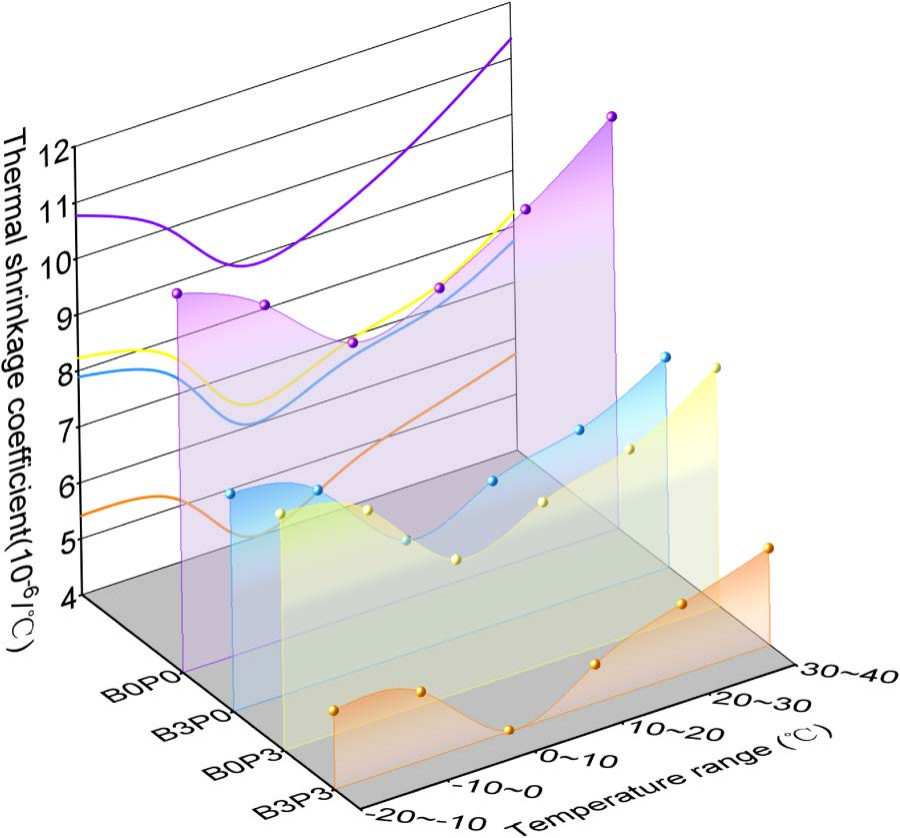
Thermal shrinkage coefficient.

Fig 12 shows that when the temperature increased, the thermal shrinkage coefficient of the basalt fiber-polycarboxylate superplasticizer cement-fly ash stabilized aeolian sand and gravel mixture (mix ratios B0P0, B0P3, B3P0, and B3P3) first decreased and subsequently increased. Overall, the thermal shrinkage changes were not significant under temperature variations. However, the addition of basalt fiber and polycarboxylate superplasticizer significantly affected the thermal behavior of the mixture. The thermal shrinkage coefficient of the single addition of basalt fiber (B3P0) and single addition of polycarboxylate superplasticizer (B0P3) decreased by at most 28.31% (20–30°C) and 31.94% (0–10°C), respectively, compared to the baseline group (B0P0). This result indicates that basalt fiber provides an internal “bridging” effect and forms an “anchoring” structure that resists deformation [42], which enhances the bonding in the cement matrix. Polycarboxylate superplasticizer increases the rate and extent of the hydration reaction and results in a denser matrix that can better resist thermal shrinkage stress. Notably, the B3P3 mixture significantly reduced thermal shrinkage coefficient by 47.96%–54.63% compared to that of B0P0 across all temperature ranges.

#### 3.2.2. Drying shrinkage.

**Fig 13:**
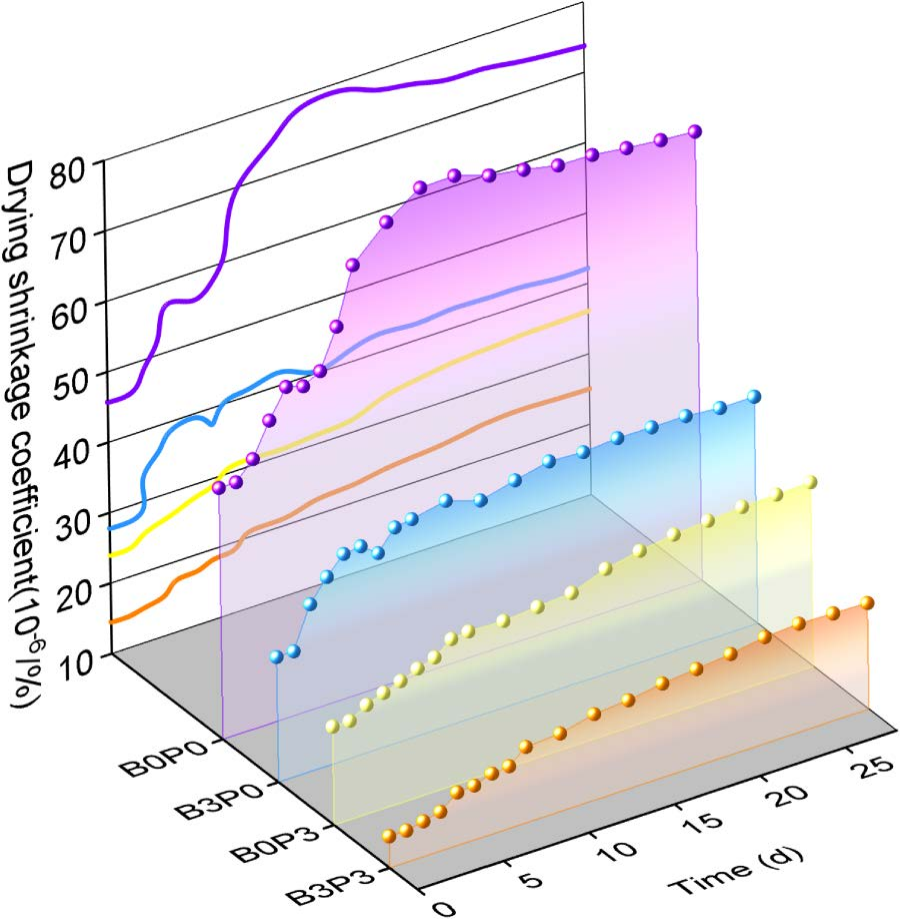
Drying shrinkage coefficient.

Fig 13 illustrates that the trend of the drying shrinkage coefficient varied among the four mix ratio groups. The drying shrinkage coefficient of the baseline mix ratio (B0P0) rapidly increased during the first 10 days and gradually stabilized after 17 days. Throughout the period of 0–14 days, it fluctuated, which indicates that the mixture without additives is highly sensitive to water loss. In contrast, the drying shrinkage coefficient of the group with the optimal content of basalt fiber (B3P0) rapidly increased in the first 5 days, subsequently slightly decreased, and gradually stabilized thereafter. This behavior suggests that in the early saturated state, a significant portion of water was consumed by fly ash and the hydration reaction. The incorporation of basalt fiber enhanced the internal structure by restricting the water movement.

The drying shrinkage coefficients for the sole addition of polycarboxylate superplasticizer (B0P3) and the combined addition of basalt fiber and polycarboxylate superplasticizer (B3P3) exhibited minimal variation throughout the test period and tended to stabilize. Both B0P3 and B3P3 demonstrated relatively low drying shrinkage strain. This is possibly because the polycarboxylate superplasticizer can reduces the initial water demand through its water-reducing and dispersing effects, which enhancing the efficiency of the cement hydration process and decreasing the amount of available water for evaporation. Furthermore, the addition of basalt fiber flattened the slope of the curve. This indicates that these modified mixtures require more water loss than the baseline group to achieve the same drying shrinkage strain. This finding underscores the effectiveness of the synergistic action in improving the resistance of the material to drying shrinkage.

**Fig 14:**
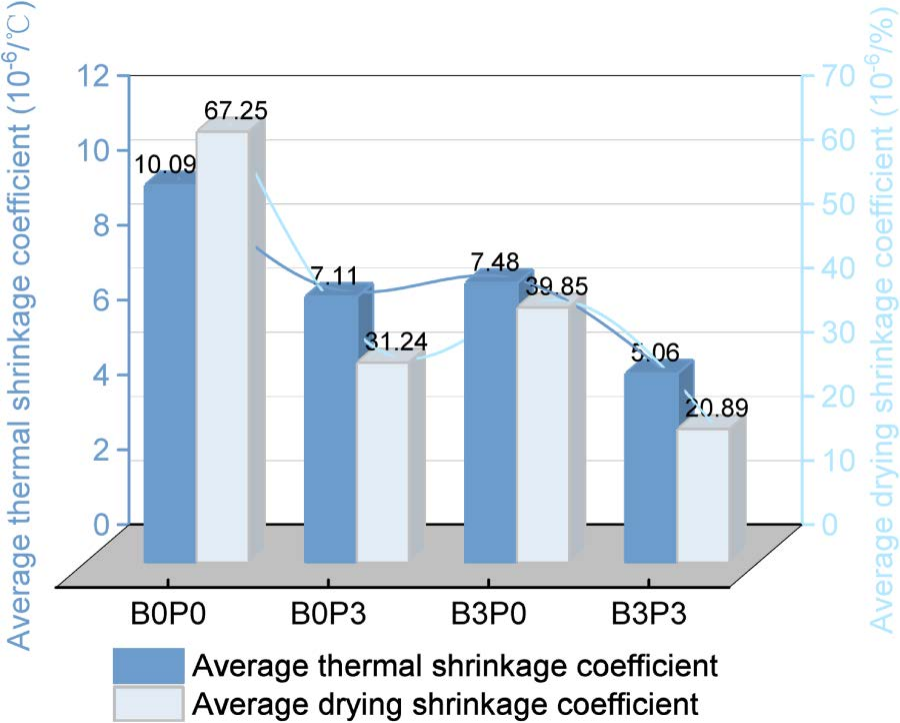
Average coefficient.

Fig 14 shows a comparison of the average thermal shrinkage coefficients and average drying shrinkage coefficients for the mixtures with mix ratios B0P0, B3P0, B0P3, and B3P3. The B3P3 group decreased the average thermal shrinkage coefficient by 49.85%, 32.35%, and 28.84% compared to the B0P0, B3P0, and B0P3 groups, respectively. Similarly, the average drying shrinkage coefficient for the B3P3 group decreased by 68.95%, 33.15%, and 47.58% compared to the B0P0, B3P0, and B0P3 groups, respectively. These findings suggest that the combination of basalt fibers and polycarboxylate superplasticizer (B3P3) significantly enhances the crack resistance of the mixture.

### 3.3. Microscopic mechanism analysis

#### 3.3.1. SEM and EDS result analysis.

**Fig 15:**
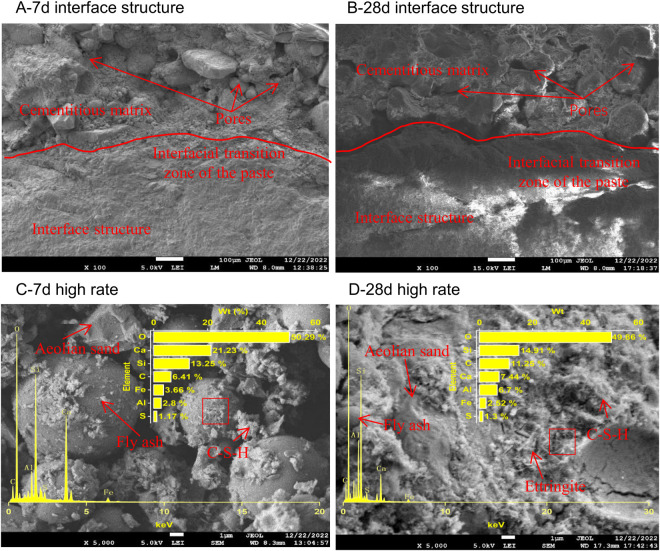
SEM image of the B0P0 mixture.

**Fig 16:**
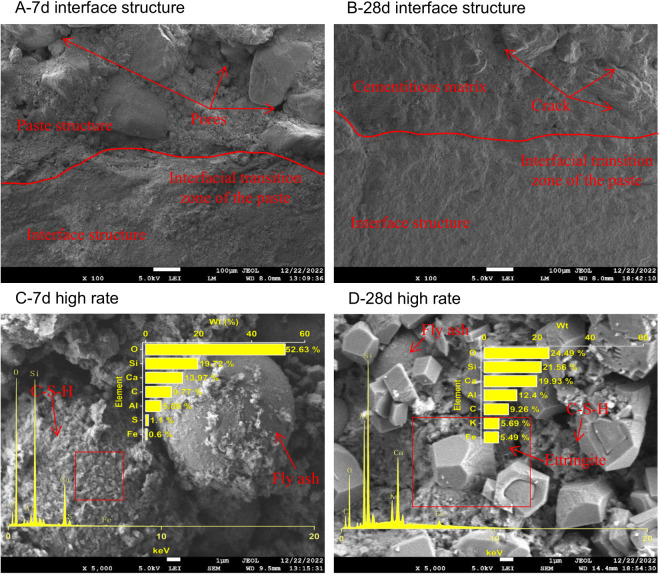
SEM image of the B0P3 mixture.

**Fig 17:**
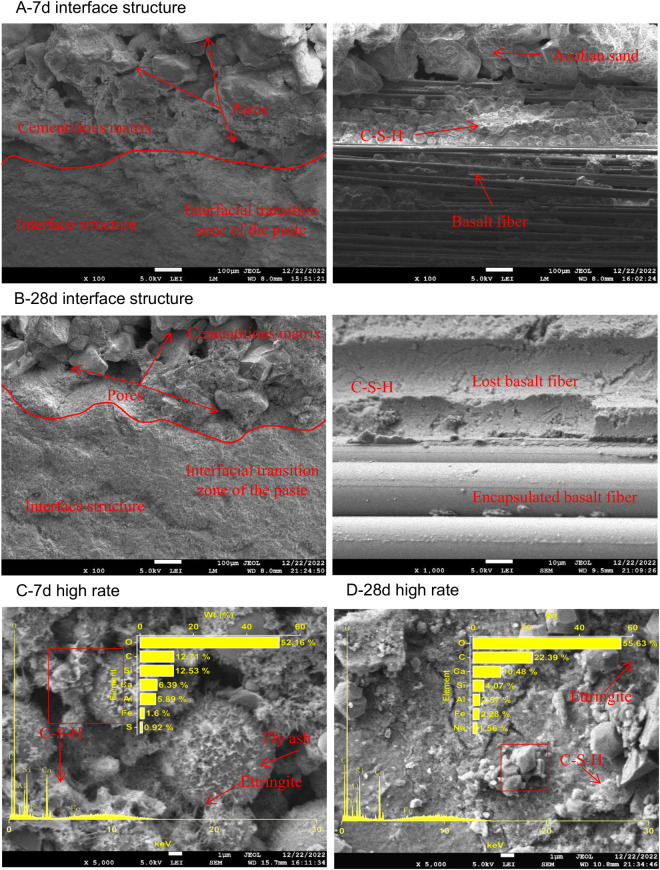
SEM image of the B3P0 mixture.

**Fig 18:**
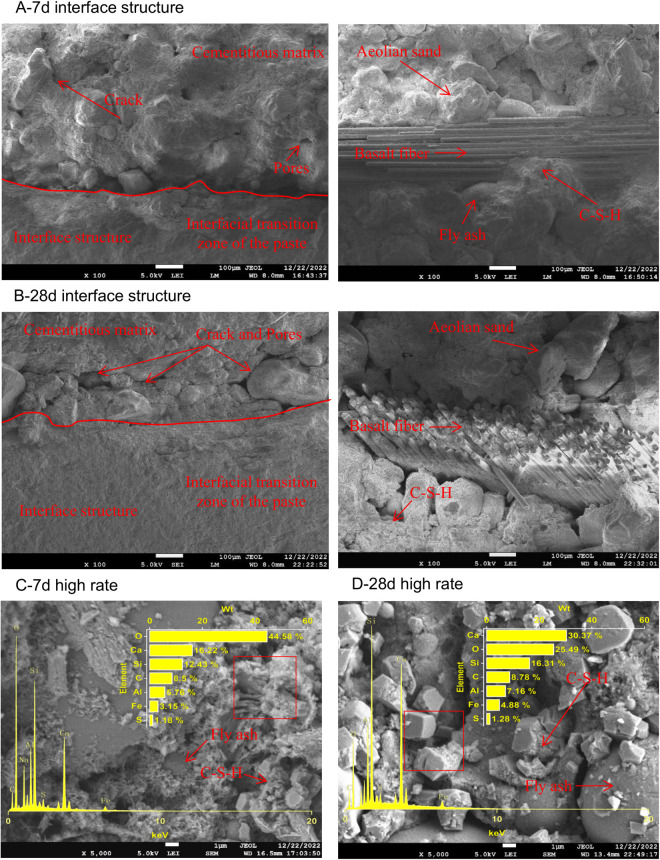
SEM image of the B3P3 mixture.

Scanning was performed on the B0P0, B0P3, B3P0, and B3P3 specimens that were cured for 7 and 28 days to observe their microstructures and the compound structures generated by hydration reactions of the mixtures, as illustrated in Figs 15–18.

As shown in Fig 15, SEM observations were conducted on the B0P0 specimens cured for 7 and 28 days. The 7-day-cured specimens exhibited wider cracks at the aggregate-paste interface, as well as more fractures and larger gel pores in their microstructure. This observation indicates incomplete hydration reactions, which resulted in fewer hydration products and an underutilization of the pozzolanic effect of fly ash. In contrast, the 28-day-cured specimens exhibited smoother paste surfaces with fewer pores, which suggests more complete hydration reactions and a denser structure of the mixture over time. At high magnification, numerous needle-like hydration products adhered to blocky gel products, and fly ash particles were enveloped by these hydration products to form a more stable three-dimensional network structure.

Fig 16 shows that the use of polycarboxylate superplasticizer significantly enhanced the microstructure of the concrete mix. After 7 days of curing, the B0P3 sample exhibited a denser microstructure, narrower cracks, and a more distinct aggregate-paste interface than the B0P0 sample. Thus, the superplasticizer contributed to a more cohesive matrix. Hydration products were observed to tightly adhere to the fly ash particles and form a network that facilitated the hydration process. By day 28, the interface transition zone in the B0P3 sample showed almost no visible cracks, the surface appeared smoother and more compact, and the porosity noticeably decreased. This result indicates a more durable and robust material, where the superplasticizer enhanced the bonding and reduced the permeability of the concrete.

SEM analysis (Fig 17) was conducted on the B3P0 samples that were cured for 7 and 28 days. The results indicate that the addition of basalt fibers led to a denser microstructure in the concrete. The interface between aggregate and paste was distinct with fewer and finer cracks than the B0P0 samples. Basalt fibers were horizontally distributed in bundles, and the gaps between the fibers were filled by cement hydration products that adhered to the fiber surfaces, which formed a stable three-dimensional network. Over time, the microstructure became increasingly dense, and the surfaces were covered by amorphous gel and layered hydration products. At a magnification of 5000 times, internal defects and pores were observed; however, these spaces were progressively occupied by blocky, reticular, needle-like, and clustered hydration products, which enhanced the overall performance of the mixture by improving the density and facilitating hydration reactions.

SEM analysis (Fig 18) was conducted on the B3P3 samples that were cured for 7 and 28 days. The synergistic effect of basalt fibers and polycarboxylate superplasticizer gradually strengthened the microstructure of the concrete mixture. At day 7, the microstructure was denser than the B0P0 samples and exhibited a more distinct and compact interface between the aggregate and the paste. The cracks on the surface of the coarse aggregates were narrower, and the porosity significantly decreased. The presence of basalt fibers played a crucial role in this densification; since the fibers unevenly fractured and were horizontally distributed, they enhanced the adhesion of the paste mixture to the hydration products that adhere to the fiber surfaces. By day 28, the interface transition zone became more refined with almost no visible cracks, which indicates a more advanced hydration process and tighter particle bonding than the 7-day samples. At this stage, the microstructure exhibited a greater variety of hydration products, including interconnected blocky and needle-like forms, which collectively contributed to a robust three-dimensional network.

Taylor [43] proposed that the Ca/Si ratio of hydration products influenced the gel formation. Specifically, when Ca/Si is less than 2.5, more gel is produced; when Ca/Si exceeds 2.5, less gel is formed. When Ca/Si is greater than 10 and both (Al + Fe)/Ca and S/Ca are less than 0.04, the primary product is calcium hydroxide (CH). Conversely, if (Al + Fe)/Ca is greater than 0.4 and S/Ca exceeds 0.25, the primary product is alumino-ferrite monosubstituted (AFm). Thus, the Ca/Si ratio serves as an indicator of hydration products in the cement interface zone [44]. A comparison of the EDS spectra results of selected areas in B0P0, B0P3, B3P0, and B3P3, as illustrated in Figs 15–18, shows that all sample regions contained fundamental elements of hydration products, including O, Ca, Si, C, Al, Fe, and S. In the early stages of curing, calcium silicate hydrate (C-S-H) emerged as one of the primary hydration products across all samples. As the curing time progressed, the formation of AFm and ettringite (AFt) became increasingly pronounced, particularly at day 28. However, there were notable differences in the chemical composition and types of hydration products among the mixtures, which were affected by the varying mix ratios and curing durations. Regarding the Ca/Si ratio, the B0P0 mixture attained its peak value at day 7 before declining by day 28. In contrast, the Ca/Si ratio in the B0P3 and B3P0 mixtures gradually increased over the curing period. The (Al + Fe)/Ca ratio significantly increased over time in the B0P0 mixture; it decreased in the B3P0 mixture, and it slightly increased in the B0P3 mixture. The morphology of hydration products also evolved over time. During the early curing stage, C-S-H predominated; however, as time progressed, the formation of AFm and AFt intensified, particularly in the 28-day samples of B0P0 and B3P3. These variations underscore the dynamic nature of internal hydration reactions and effects of different mix ratios on the development of hydration products and microstructure.

#### 3.3.2. XRD result analysis.

Phase analysis was conducted on the samples with curing periods of 7 days (B0P0, B0P3, B3P0, B3P3) and 28 days (B0P0, B0P3, B3P0, B3P3).

**Fig 19:**
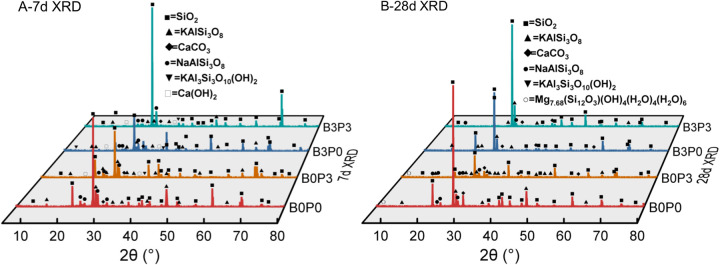
XRD spectrum analysis.

As illustrated in Fig 19, during the 7-day curing period, the identified primary phases in the control group B0P0 were KAlSi_3_O_8_, CaCO_3_, and NaAlSi_3_O_8_, which indicate standard hydration reactions. In the group with only polycarboxylate superplasticizer (B0P3) and the group with only basalt fiber (B3P0), new crystalline phases of KAl_3_Si_3_O_10_(OH)_2_ and Ca(OH)_2_ were detected compared to those in B0P0. Thus, the polycarboxylate superplasticizer accelerated the hydration reaction by enhancing the interaction and reaction among cement particles, which increased the quantity of KAl_3_Si_3_O_10_(OH)_2_ and Ca(OH)_2_ at 7 days and improved the early strength of the mixture. The incorporation of basalt fiber facilitated the formation of new crystals due to its silicate and aluminate content, which served as nucleation sites for new mineral phases and enhanced the mechanical properties of the mixture. In the group with both polycarboxylate superplasticizer and basalt fiber (B3P3), the synergistic interaction of the two additives formed a more diverse array of mineral phases and further enhanced the mechanical properties of the mixture.

At the conclusion of the 28-day curing period, the identified primary phases in the control group B0P0 were KAlSi_3_O_8_, CaCO_3_, NaAlSi_3_O_8_, and Mg_7.68_(Si_12_O_3_)(OH)_4_(H_2_O)_4_(H_2_O)_6_. The B0P3 group, which contained only polycarboxylate superplasticizer, exhibited no differences compared to the B0P0 group, since both groups contained Mg_7.68_(Si_12_O_3_)(OH)_4_(H_2_O)_4_(H_2_O)_6_. Thus, the superplasticizer delayed the overall hydration process and resulted in a similar phase composition to that of B0P0 due to incomplete reactions. However, the crystals of KAl_3_Si_3_O_10_(OH)_2_ and Ca(OH)_2_ that were observed at 7 days had completely reacted to form CaCO_3_ by day 28, which suggests that the superplasticizer enhanced the hydration of calcium compounds and the strength of the mixture. In the B3P0 group, which contained only basalt fiber, the absence of NaAlSi_3_O_8_ and Mg_7.68_(Si_12_O_3_)(OH)_4_(H_2_O)_4_(H_2_O)_6_ indicates that the hydration reaction was nearing completion, so there was no significant strength increase after 28 days. In the group that included both additives (B3P3), the interaction between polycarboxylate superplasticizer and basalt fiber formed a more diverse array of mineral phases and further enhanced the mechanical properties of the mixture.

**Fig 20:**
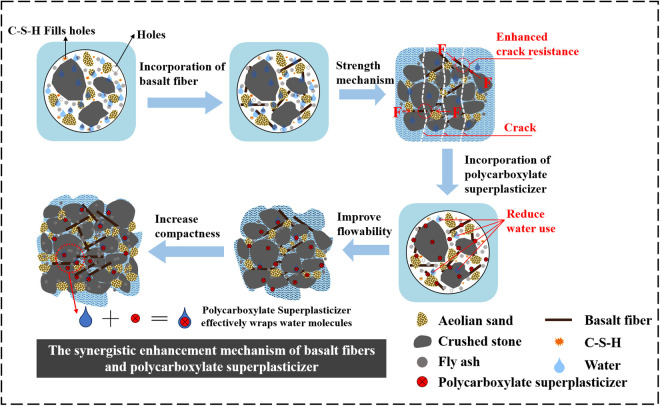
The synergistic enhancement mechanism of basalt fibers and poly carboxylate superplasticizer.

Fig 20 illustrates the reaction mechanism by which polycarboxylate superplasticizer and basalt fibers optimize the aeolian sand-gravel base. Firstly, the addition of basalt fibers provides a “reinforcement” effect, where the friction between the hydration products and the aggregate reduces cracks caused by holes. Secondly, with the simultaneous addition of a polycarboxylate superplasticizer, its molecules adsorb onto the surface of cement particles through electrostatic repulsion and steric hindrance effects, enhancing the dispersion of the cement particles. This dispersion improves the flowability of the paste, increases the contact area between the cement particles and water, and reduces the amount of water needed in the mixture. Furthermore, the polycarboxylate superplasticizer decreases porosity and inhibits segregation and bleeding, resulting in a denser structure. These improvements, combined with the interlacing of basalt fibers, form a three-dimensional network structure, significantly enhancing the mechanical strength and crack resistance of the aeolian sand-gravel base. This is the reason for the superior performance of the combined use of water-reducing agents and basalt fibers in strengthening the mechanical and crack resistance properties of the aeolian sand-gravel base.

### 3.4. Discussion

At the macroscopic scale, the compressive and splitting tensile strengths of mixtures B0P3, B3P0, and B3P3 showed respective increases of 14%, 16%, and 33%, and 20%, 31%, and 52%, compared to the control group B0P0. This clearly demonstrates a trend of strength improvement as follows: dual admixture (B3P3)> basalt fiber only (B3P0)> superplasticizer only (B0P3). These improvements can be partially attributed to the enhanced microstructural features observed at the microscale. In particular, the SEM images of B0P3 revealed a denser matrix and a more refined microstructure compared to B0P0, which aligns with the EDS analysis at 7 days, where the Ca/Si ratio was found to be 0.7 in B0P3 versus 1.6 in B0P0. The lower Ca/Si ratio indicates a denser and more polymerized C–S–H gel, contributing to improved strength. In B3P0, the Ca/Si ratio further decreased to 0.5, suggesting that the incorporation of basalt fiber accelerates the hydration process, resulting in rapid early strength gain. However, in the dual-admixture system (B3P3), the Ca/Si ratio was 1.3, which reflects a moderated hydration rate due to the presence of polycarboxylate superplasticizer. This controlled hydration allows for the formation of a more uniform and stable microstructure, while also facilitating better fiber dispersion and reduced agglomeration. In particular, the SEM images revealed a visibly improved interfacial transition zone (ITZ) between the fiber and the cement matrix, especially in B3P3. This multiscale correlation indicates that the distribution of fibers and the quality of the fiber–matrix interface at the microscale play a crucial role in determining the overall mechanical performance. The synergistic effect of fiber reinforcement and hydration regulation contributes significantly to the superior strength observed in the dual-admixture system.

Furthermore, the early-age compressive strength of specimens containing higher dosages of polycarboxylate superplasticizers exhibited a slight delay compared to the control. This suggests a possible retardation effect on cement hydration. Similar phenomena have been widely reported in the literature. For instance, Zhang et al. [45] demonstrated that polycarboxylate superplasticizers with varying molecular architectures significantly delayed the early hydration of cement and reduced early-age strength development. The observed strength trends in our study are consistent with these findings. In addition, Fang et al. [46] reported that the strong adsorption of PCE molecules on cement particle surfaces hinders the dissolution of clinker phases and suppresses the nucleation and growth of C–S–H gel. This mechanism provides a plausible explanation for the reduced early hydration activity observed in our high-PCE mixtures. However, the long-term strength was not adversely affected, indicating that the hydration process was only temporarily delayed and that sufficient hydration occurred over time.

## Conclusions

To enhance the road performance of aeolian sand cement-stabilized bases, additives such as basalt fibers and polycarboxylate superplasticizer were studied. Dosage variations of basalt fibers (0–0.15%) and superplasticizer (0–1.5%) were tested for their effects on 7-, 14-, and 28-day UCS and STS. The optimal basalt fiber length was 12 mm. Selected proportions underwent crack resistance tests via drying and thermal shrinkage assessments. The strength mechanisms were elucidated via XRD, SEM, and EDS. The conclusions are as follows:

- With increased curing time, all mix proportions increased in UCS. At 7, 14, and 28 days, the mixes with only polycarboxylate superplasticizer had 9.33%, 13.95%, and 14% higher UCS than the control group, respectively, and the mixes with only basalt fiber had 8%, 15.12%, and 16% higher UCS than the control group, respectively. The combination of both additives exhibited the greatest increase in UCS with improvements of 33.00%, 14%, and 16% compared to the control group, the mix with only polycarboxylate superplasticizer, and the mix with only basalt fiber, respectively, at 28 days.
- The STS of all mix proportions increased with longer curing times. At 7, 14, and 28 days, the STS improved by 21.82%, 31.15%, and 16%, respectively, with only polycarboxylate superplasticizer and by 12.73%, 19.67%, and 18.67%, respectively, with only basalt fiber compared to the control group. The combined additives resulted in the highest increase in STS with improvements of 52.00%, 28.09%, and 31.51% compared to the control group, the mix with only polycarboxylate superplasticizer, and the mix with only basalt fiber, respectively.
- The highest UCS and STS of the mix were achieved with a basalt fiber length of 12 mm at optimal content B3. Thus, the best mix proportion was the combination of 0.1% volume fraction of 12-mm basalt fiber and 1.0% mass fraction of polycarboxylate superplasticizer.
- The thermal shrinkage coefficient of all mix proportions first decreased and subsequently increased with increasing temperature. Compared to the control group, the coefficients decreased by 29.53% and 25.87% with only polycarboxylate superplasticizer and basalt fiber, respectively. The combination of both additives decreased the coefficient by 49.85%, 32.35%, and 28.84% compared to the control group, the mix with only polycarboxylate superplasticizer, and the mix with only basalt fiber, respectively.
- The drying shrinkage coefficient of all mix proportions first increased and subsequently stabilized over time. The coefficients decreased by 53.55% and 40.74% with only polycarboxylate superplasticizer and basalt fiber, respectively, compared to that of the control group. The combination of both additives showed minimal variation in drying shrinkage coefficient throughout the testing period, i.e., the samples exhibited a more stable overall trend. Reductions of 68.94%, 33.15%, and 47.58% were observed compared to the control group, the mix with only polycarboxylate superplasticizer, and the mix with only basalt fiber, respectively.
- When the curing period was extended from 7 days to 28 days, the microstructure of mixes with only polycarboxylate superplasticizer and basalt fiber became denser than that of the control group. This improvement is attributed to the superplasticizer enhancing the hydration process and basalt fibers forming a stable three-dimensional network through the hydration products that adhere to their surface. The combination of both additives results in the most significant densification and performance enhancement.
- Based on current research findings, future studies will focus on the long-term performance evaluation to assess the durability and aging effects on the strength and crack resistance of modified cement-stabilized base under various environmental conditions. Additionally, the proportions of basalt fiber and polycarboxylate superplasticizer will be further optimized to better balance the cost and performance.

## Supporting information

S1 File Supporting tables including S1 Table (UCS and STS for all mix ratios), S2 Table (Mechanical strength values of the different fiber lengths and curing durations), S3 Table (Test results of thermal shrinkage strain), S4 Table (Test results of thermal shrinkage coefficient), S5 Table (Cumulative water loss rate and drying shrinkage), and S6 Table (Cumulative drying shrinkage strain and drying shrinkage coefficient). (PDF)
