# Supplementary material for: Synergistic effects and mechanisms of basalt fibers and polycarboxylate superplasticizer on cement–fly ash stabilized aeolian sand and crushed stones
Source: PLoS One. 2025 Jul 15;20(7):e0327351. doi: 10.1371/journal.pone.0327351 (PMC12262880; doi:10.1371/journal.pone.0327351)
Supplement: S1 File — (PDF) [file pone.0327351.s001.pdf]

Supporting information

S1 Table

Mixture ratios	UCS/MPa			STS/MPa		
	7d	14d	28d	7d	14d	28d
B0P0	7.5	8.6	10.0	0.55	0.61	0.75
B0P1	7.8	9.0	10.4	0.57	0.65	0.83
B0P2	8.1	9.7	10.6	0.59	0.68	0.85
B0P3	8.2	9.9	11.4	0.62	0.73	0.89
B0P4	8.1	9.6	10.8	0.56	0.65	0.79
B0P5	7.6	8.8	9.7	0.53	0.61	0.73
B1P0	7.6	8.8	10.3	0.57	0.66	0.80
B1P1	8.0	9.4	10.5	0.60	0.71	0.87
B1P2	8.2	9.9	10.8	0.63	0.75	0.90
B1P3	8.4	10.3	11.9	0.66	0.79	0.92
B1P4	8.1	9.7	11.0	0.64	0.76	0.84
B1P5	7.7	9.2	10.2	0.55	0.64	0.77
B2P0	7.7	9.1	11.0	0.64	0.75	0.83
B2P1	8.2	10.0	11.1	0.67	0.80	0.89
B2P2	8.5	10.4	11.7	0.69	0.82	0.92
B2P3	8.6	10.9	12.4	0.73	0.90	1.06
B2P4	8.3	10.2	11.1	0.68	0.81	0.86
B2P5	7.8	9.4	10.8	0.57	0.67	0.81
B3P0	8.1	9.8	11.6	0.67	0.80	0.87
B3P1	8.4	10.3	12.1	0.68	0.83	0.92
B3P2	8.6	11.0	12.3	0.69	0.85	0.97
B3P3	8.9	11.4	13.3	0.76	0.96	1.14
B3P4	8.5	10.5	12.0	0.68	0.83	0.88
B3P5	8.0	9.6	10.8	0.62	0.74	0.85
B4P0	7.9	9.2	11.0	0.58	0.69	0.77
B4P1	8.0	9.6	11.3	0.63	0.77	0.84
B4P2	8.3	10.2	11.4	0.66	0.80	0.90
B4P3	8.7	11.0	12.5	0.68	0.83	0.97
B4P4	7.9	9.5	10.7	0.56	0.67	0.78
B4P5	7.5	8.7	10.5	0.54	0.63	0.74
B5P0	7.8	9.1	10.6	0.51	0.58	0.67
B5P1	7.9	9.5	11.1	0.53	0.61	0.74
B5P2	8.2	9.9	11.2	0.56	0.68	0.81
B5P3	8.3	10.2	11.7	0.59	0.72	0.86
B5P4	7.8	9.4	10.5	0.54	0.63	0.74
B5P5	7.4	8.4	10.3	0.51	0.59	0.64

S2 Table.

Mixture ratios	Fiber lengths/mm	UTS/MPa			STS/MPa		
		7d	14d	28d	7d	14d	28d
B3P0	6	7.6	8.7	10.5	0.53	0.60	0.77
	9	7.8	9.2	10.6	0.60	0.70	0.82
	12	8.2	9.9	11.4	0.62	0.73	0.89
	15	7.5	8.6	9.8	0.60	0.71	0.81
	18	7.2	8.2	9.6	0.50	0.58	0.70
B3P3	6	8.0	9.8	12.5	0.67	0.81	0.87
	9	8.5	10.6	12.9	0.72	0.87	0.95
	12	8.9	11.4	13.3	0.76	0.96	1.13
	15	8.0	9.6	11.6	0.62	0.77	0.86
	18	7.8	9.2	10.8	0.60	0.71	0.80

S3 Table.

Mixture ratios	Thermal shrinkage strain/10^{-6}						Cumulative thermal shrinkage strain/10^{-6}
	40-30°C	30-20°C	20-10°C	10-0°C	0--10°C	-10--20°C	
B0P0	113.5	102.1	93.2	88.6	100.5	107.7	605.6
B0P3	77.5	69.7	65.7	60.3	74.4	78.9	426.5
B3P0	82.6	73.2	68.9	63.8	77.8	82.3	448.6
B3P3	57.3	52.5	46.9	40.2	52.3	54.1	303.3

S4 Table.

Mixture ratios	Thermal shrinkage coefficient/$10^{-6}\cdot°C^{-1}$						Average thermal shrinkage coefficient $10^{-6}\cdot°C^{-1}$
	40-30°C	30-20°C	20-10°C	10-0°C	0--10°C	-10--20°C	
B0P0	11.35	10.21	9.32	8.86	10.05	10.77	10.09
B0P3	7.75	6.97	6.57	6.03	7.44	7.89	7.11
B3P0	8.26	7.32	6.89	6.38	7.78	8.23	7.48
B3P3	5.73	5.25	4.69	4.02	5.23	5.41	5.06

S5 Table.

Curing age/d	Cumulative water loss rate/%				Cumulative drying shrinkage/μm			
	B0P0	B0P3	B3P0	B3P3	B0P0	B0P3	B3P0	B3P3
1	0.93	0.73	0.81	0.69	17	7	9	4
2	1.92	1.67	1.56	1.50	37	17	21	9
3	2.98	2.75	2.45	2.41	63	29	36	15
4	3.74	3.58	3.38	3.00	85	39	53	21
5	4.59	4.17	4.18	3.54	103	47	66	25
6	4.96	4.40	4.51	3.77	114	51	68	28
7	5.28	4.63	4.81	3.99	133	57	78	30
8	5.43	4.75	4.95	4.10	154	59	81	34

10	5.67	4.93	5.19	4.28	171	61	87	36
12	5.88	5.03	5.39	4.40	185	63	87	39
14	6.19	5.28	5.65	4.66	195	67	94	42
16	6.35	5.45	5.86	4.83	196	73	100	45
18	6.48	5.59	6.02	4.95	198	77	102	47
20	6.60	5.69	6.14	5.05	199	80	105	49
22	6.71	5.78	6.24	5.09	202	82	107	51
24	6.80	5.86	6.33	5.13	203	84	108	52
26	6.88	5.91	6.40	5.16	204	85	108	52
28	6.95	5.95	6.46	5.19	205	86	109	52

S6 Table

Curing age/d	Drying shrinkage strain/10^{-6}				Drying shrinkage coefficient/$10^{-6}\cdot\%^{-1}$			
	B0P0	B0P3	B3P0	B3P3	B0P0	B0P3	B3P0	B3P3
1	42.50	17.5	22.5	10.0	45.70	23.97	27.78	14.49
2	92.50	42.5	52.5	22.5	48.18	25.45	33.65	15.00
3	157.50	72.5	90.0	37.5	52.85	26.36	36.73	15.56
4	212.50	97.5	132.5	52.5	56.82	27.23	39.20	17.50
5	257.50	117.5	165.0	62.5	56.10	28.18	39.47	17.66
6	285.00	127.5	170.0	70.0	57.46	28.98	37.69	18.57
7	332.50	142.5	195.0	75.0	62.97	30.78	40.54	18.80
8	385.00	147.5	202.5	85.0	70.90	31.05	40.91	20.73
10	427.50	152.5	217.5	90.0	75.40	30.93	41.91	21.03
12	462.50	157.5	217.5	97.5	78.66	31.31	40.35	22.16
14	487.50	167.4	235.0	105.0	78.76	31.70	41.59	22.53
16	490.00	182.5	250.0	112.5	77.17	33.49	42.66	23.29
18	495.00	192.5	255.0	117.5	76.39	34.44	42.36	23.74
20	497.50	199.9	262.5	122.5	75.38	35.13	42.75	24.26
22	505.00	205.0	266.3	127.5	75.26	35.47	42.67	25.05
24	507.50	210.0	270.0	130.0	74.63	35.84	42.65	25.34
26	510.00	212.5	270.0	130.0	74.13	35.96	42.19	25.19
28	512.50	215.0	272.5	130.0	73.74	36.13	42.18	25.05
